# Association between consumption of flavonol and its subclasses and chronic kidney disease in US adults: an analysis based on National Health and Nutrition Examination Survey data from 2007–2008, 2009–2010, and 2017–2018

**DOI:** 10.3389/fnut.2024.1399251

**Published:** 2024-06-18

**Authors:** Peijia Liu, Leile Tang, Guixia Li, Xiaoyu Wu, Feng Hu, Wujian Peng

**Affiliations:** ^1^Department of Nephrology, Shenzhen Third People’s Hospital, The Second Affiliated Hospital of Southern University of Science and Technology, Shenzhen, China; ^2^Department of Cardiology, The Third Affiliated Hospital of Sun Yat-sen University, Guangzhou, China

**Keywords:** flavonol, isorhamnetin, chronic kidney disease, NHANES, estimated glomerular filtration rate, urine albumin-to-creatinine ratio

## Abstract

**Background:**

There is little research on the relationship between flavonol consumption and chronic kidney disease (CKD). This study aimed to examine the link between flavonol consumption and the risk of CKD among US adults, using data from the 2007–2008, 2009–2010 and 2017–2018 National Health and Nutrition Examination Survey (NHANES).

**Methods:**

A cross-sectional approach was used, drawing on data from three NHANES cycles. The flavonol consumption of the participants in this study was assessed using a 48 h dietary recall interview. CKD was diagnosed based on an estimated glomerular filtration rate below 60 mL/min/1.73 m^2^ or a urine albumin-to-creatinine ratio of 30 mg/g or higher.

**Results:**

Compared to the lowest quartile of flavonol intake (Q1), the odds ratios for CKD were 0.598 (95% CI: 0.349, 1.023) for the second quartile (Q2), 0.679 (95% CI: 0.404, 1.142) for the third quartile (Q3), and 0.628 (95% CI: 0.395, 0.998) for the fourth quartile (Q4), with a *p* value for trend significance of 0.190. In addition, there was a significant trend in CKD risk with isorhamnetin intake, with the odds ratios for CKD decreasing to 0.860 (95% CI: 0.546, 1.354) in the second quartile, 0.778 (95% CI: 0.515, 1.177) in the third quartile, and 0.637 (95% CI: 0.515, 1.177) in the fourth quartile (*p* for trend = 0.013).

**Conclusion:**

Our analysis of the NHANES data spanning 2007–2008, 2009–2010, and 2017–2018 suggests that high consumption of dietary flavonol, especially isorhamnetin, might be linked to a lower risk of CKD in US adults. These findings offer new avenues for exploring strategies for managing CKD.

## Introduction

Chronic kidney disease (CKD) is a major concern for public health globally, which demands immediate and widespread attention worldwide. Data sourced from the Chronic Kidney Disease Epidemiology Collaboration (CKD-EPI) indicate that around 700 million people worldwide are impacted by CKD, and this number exhibits a consistent upward trend (1). Similarly, the United States (US) is facing this health challenge. Data from the US Centers for Disease Control and Prevention show that about 37 million people in the US, or roughly 15% of its total population, are dealing with CKD (2). The treatment modalities for CKD primarily include lifestyle modifications and the implementation of a low-salt, low-protein diet, along with the management of proteinuria, blood pressure, blood glucose, uric acid, and lipid levels (3–5). Despite these measures, the therapeutic options available for CKD remain somewhat limited.

Flavonol is a polyphenolic compound widely found in tea, berries, and onions, and belongs to the class of flavonoids. These compounds display a diverse range of biological activities including antioxidant, anti-inflammatory, antifibrotic, and immunomodulatory, all of which appear to contribute to improved health outcomes (6–9). Evidence suggests that high flavonol consumption may confer benefits to patients suffering from cardiovascular diseases (10, 11), neurodegenerative disorders (12, 13), diabetes (14), hyperlipidemia (15), metabolic syndrome (16, 17), and those in a debilitating state (18). Preliminary research indicates that flavanol and its subclasses can protect the kidneys through various mechanisms (19–23). Although clinical evidence regarding the renal benefits of flavanol is still limited, some clinical studies have indicated that flavonol may enhance endothelial function in populations with end-stage renal disease, diabetes, and even in healthy individuals (24–26). As the pathogenesis of CKD is partly associated with endothelial damage, it is plausible that flavonol may provide renal benefits to CKD patients (27). Current research also reveals that the consumption of foods rich in flavonoids is associated with a lower incidence of diabetic nephropathy (28). Therefore, increasing flavonol intake may potentially benefit individuals with CKD. However, clinical evidence directly linking flavonol consumption to reduced CKD incidence is limited. Thus, the potential health benefits of flavonol intake for CKD patients warrant further investigation to establish a more definitive understanding of this relationship.

Based on the aforementioned findings, we hypothesize that high intake of flavonol may be associated with a lower risk of CKD. In this study, we examined this association by analyzing cross-sectional data from the National Health and Nutrition Examination Survey (NHANES) database. Recognizing the potential for variability in the biological activities of different flavonol types, our study also examined the relationship between the incidence of CKD and four flavonol subclasses, namely isorhamnetin, kaempferol, myricetin, and quercetin.

## Methods

### Study design and study population

The NHANES database is a publicly available compendium, comprising participant data collected biennially through a multistage, stratified sampling method. This compendium encompasses a diverse range of data categories, including demographic profiles, questionnaire responses, dietary intakes, physical examinations, and laboratory evaluations. Using specific weighting protocols, researchers can adjust these data to make the resultant dataset representative of the broader US population (29).

This study used a cross-sectional design approach, incorporating data from three distinct phases of the ongoing NHANES, specifically the cycles spanning 2007–2008, 2009–2010, and 2017–2018. After performing the data weighting procedures, the subset extracted for further analytical examination included individuals aged 20 years or older, with available data on estimated glomerular filtration rate (eGFR) and proteinuria, as well as on the intake of flavonol and its subclasses.

### Consumption of flavonol and its subclasses

The determination of daily nutrient intake from food sources was achieved through a complex methodology (16, 17, 30). Briefly, this segment of data collection was organized under the auspices of the US Department of Agriculture (USDA). To ensure precise acquisition of dietary intake data, trained interviewers used the USDA-developed Automated Multiple-Pass Method (AMPM). This methodology encompasses a comprehensive set of standardized queries tailored for specific food items, along with a wide array of potential response options. The process involved an initial 24 h dietary recall (Day 1), conducted face-to-face at the NHANES Mobile Examination Center, followed by a secondary recall (Day 2) completed by phone between 3 to 10 days later. The AMPM has been extensively validated in research studies and demonstrated to be an effective approach for measuring group energy intake and adult sodium intake (31, 32). The flavonol intake was calculated by multiplying the flavonol content in each food item by the consumption frequency obtained from the food frequency questionnaire. To accurately match foods containing flavonol and determine their flavonol content, food codes from the Food and Nutrient Database for Dietary Studies (FNDDS) were used. Specifically, the NHANES 2007–2008 dataset used FNDDS version 4.1 food codes, whereas the NHANES 2009–2010 and 2017–2018 datasets used FNDDS version 5.0 food codes (16, 17, 30). In this study, the aggregate flavonol intake during Day 1 and Day 2 was use as a metric to measure the flavonol consumption of each participant. Additionally, the flavonol compounds included: isorhamnetin, kaempferol, myricetin, and quercetin.

### Study outcomes

A solid-phase fluorescence immunoassay was performed to determine the levels of urinary albumin in human samples (33). The Jaffe rate method was used to measure the concentrations of creatinine in both serum and urine (34). The eGFR was calculated using the 2012 CKD-EPI equation with every serum creatinine measurement (35). The primary endpoint of this study was CKD, defined as an eGFR of less than 60 mL/min/1.73 m^2^ and/or a urine albumin-to-creatinine ratio (UACR) exceeding 30 mg/g (3, 4). The secondary endpoints were separately identified as proteinuria (UACR exceeding 30 mg/g) and a decline in eGFR (eGFR less than 60 mL/min/1.73 m^2^).

### Covariates

This study considered multiple demographic covariates, including age, sex (male, female), race/ethnicity (non-Hispanic white, non-Hispanic black, Mexican American, and others), education level (high school or above, middle school or below), and economic status. Economic status was measured by the poverty income ratio (PIR), with a PIR less than 1 indicating poverty and PIR equal to or greater than 1 indicating non-poverty (36). Physical examinations included height, weight, body mass index (BMI), diastolic blood pressure (DBP), and systolic blood pressure (SBP). The BMI was calculated as weight in kilograms divided by height in meters squared, with a BMI of 25 kg/m^2^ or greater defined as overweight or obese. Blood pressure was measured by trained staff, typically as an average of three readings. Personal habits included smoking history and physical activity. Smoking status includes never, former, and current smokers. “Never” is defined as having smoked fewer than 100 cigarettes in their lifetime. “Former” refers to individuals who have smoked at least 100 cigarettes in their lifetime but do not smoke at all currently. “Current” indicates individuals who have smoked at least 100 cigarettes in their lifetime and continue to smoke either on some days or every day. The physical activity was assessed in metabolic equivalent of task (MET) minutes per week and categorized into two groups based on whether it exceeded 600 MET minutes per week (37). Energy intake was the sum of calories consumed over the 2 days. Laboratory tests included total cholesterol, fasting lipids, fasting glucose, uric acid, and hemoglobin. The accuracy of the methods used to detect these substances was ensured by strict quality control procedures. Among the assessed complications were: hyperuricemia, defined as uric acid levels exceeding 360 μmol/L in women or 420 μmol/L in men (38); dyslipidemia, characterized by any of the following: triglyceride over 150 mg/dL, total cholesterol over 200 mg/dL, low-density lipoprotein above 130 mg/dL, high-density lipoprotein below 40 mg/dL for men or 50 mg/dL for women, or the use of lipid-lowering drugs (39); diabetes, diagnosed by one or more of the following criteria: glycated hemoglobin greater than 6.5%, fasting glucose above 7.0 mmol/L, random glucose over 11.1 mmol/L, postprandial glucose exceeding 11.1 mmol/L after 2 hours, use of hypoglycemic medications, or a clinical diagnosis (40); hypertension, defined by a systolic blood pressure higher than 140 mmHg or diastolic blood pressure over 90 mmHg, the use of antihypertensive drugs, or a clinical diagnosis (41).

### Statistical analyses

**Figure 1:**
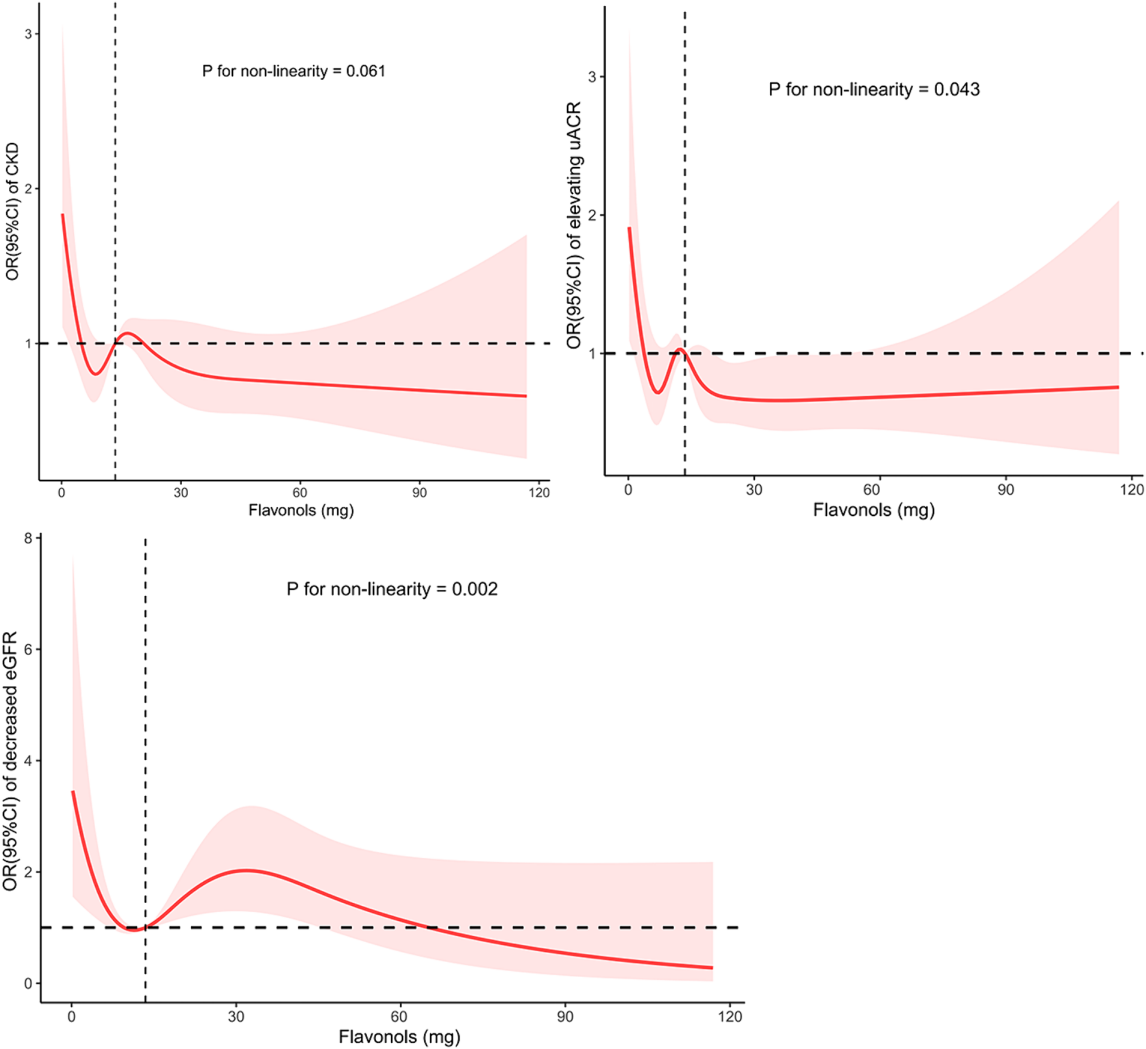
The non-linear trend between the intake of flavonol and CKD risk, proteinuria and decreased eGFR using a restricted cubic spline. Data are presented as OR (95%CI) (*y*-axis) and level of flavonol (mg per 48 h) after adjusted for age, sex, body mass index, ethnicity, smoke status, physical activity status, systolic blood pressure, diastolic blood pressure, energy intake, uric acid, fasting glucose, cholesterol, fasting triglyceride, hyperuricemia, dyslipidemia, hypertension, and diabetes mellitus. OR, odds ratio; CI, confidence interval; eGFR, estimated glomerular filtration; CKD, chronic kidney disease.

The data from the NHANES database were collected using a complex, multilevel stratified sampling method. Thus, to ensure that the analysis reflects the broader demographics of the US, all collected data were subjected to a weighting procedure prior to analysis. Continuous variables are expressed as mean values with 95% confidence intervals (CIs), and categorical variables are expressed as percentages with their 95% CIs in Table 1. Statistical differences between CKD and non-CKD groups were evaluated using the rank-sum test for continuous variables and the chi-square test for categorical variables in Table 1. Based on the differences between the CKD and non-CKD populations, as well as clinical experience, we selected the appropriate variables to serve as calibration variables for subsequent analyses, which are specifically presented in the results section. The consumption of flavonol was divided into four distinct groups, each representing a quartile, namely the first quartile (Q1), the second quartile (Q2), the third quartile (Q3), and the fourth quartile (Q4). This study investigated the correlation between flavonol intake and CKD risk using a weighted multivariable logistic regression model, with an emphasis on trend analysis. The results were presented in Tables 2, 3. The dose–response relationship between flavonol consumption and CKD risk was analyzed using restricted cubic splines (RCS), as shown in Figure 1. In subgroup analyses, the association between flavonol intake and CKD risk was examined across various subgroups, complemented by interaction tests. The results were presented in Table 4. The relationships between specific flavonol compounds (isorhamnetin, kaempferol, myricetin, and quercetin) and CKD risk were analyzed using a weighted multivariable logistic regression method. The results were presented in Table 5. Statistical significance was assessed using a two-tailed *p*-value threshold of less than 0.05. All statistical evaluations were performed using the R software version 4.3.0 (R Development Core Team, University of Auckland, Auckland City, NZ).

**Table 1:** Characteristics of CKD and non-CKD in the US population.

Characteristics	Non-CKD group	CKD group	*p* value
Demography			
Age (years)	45.2 (44.6, 45.8)	60.9 (59.9,62.0)	<0.001
Age group			<0.001
20 ~ 59	79.70 (78.25, 81.15)	39.79 (36.88, 42.70)	
60~	20.30 (18.85, 21.75)	60.21 (57.30, 63.12)	
Sex			0.002
Female	51.96 (50.78, 53.15)	57.00 (54.03, 59.97)	
Male	48.04 (46.85, 49.22)	43.00 (40.03, 45.97)	
Ethnicity			<0.001
White	67.10 (63.34, 70.86)	70.14 (66.16, 74.12)	
Black	10.60 (8.83, 12.37)	12.34 (10.08, 14.61)	
Mexican	8.88 (6.82, 10.94)	7.55 (5.82, 9.29)	
Others	13.42 (11.47, 15.38)	9.97 (7.69, 12.25)	
Education			0.486
High school or above	55.17 (52.87, 57.47)	56.28 (53.19, 59.38)	
Others	44.83 (42.53, 47.13)	43.72 (40.62, 46.81)	
Poverty income ratio			0.834
No	86.36 (85.02, 87.69)	86.18 (84.26, 88.10)	
Yes	13.64 (12.31, 14.98)	13.82 (11.90, 15.74)	
Physical examination and personal history			
SBP (mmHg)	119.9 (119.3, 120.4)	131.2 (129.6, 132.8)	<0.001
DBP (mmHg)	71.2 (70.5, 71.9)	69.5 (68.6, 70.5)	<0.001
Height (cm)	168.9 (168.6, 169.2)	165.9 (165.3, 166.5)	<0.001
Weight (kg)	82.8 (82.1, 83.5)	85.0 (83.3, 86.6)	0.017
BMI (kg/m^2^)	28.9 (28.7, 29.2)	30.7 (30.1, 31.2)	<0.001
Energy intake (kcal)	4,352 (4,188, 4,416)	3,806 (3,694, 3,920)	<0.001
Overweight and obesity			<0.001
No	29.69 (27.95, 31.43)	22.74 (20.28, 25.19)	
Yes	70.31 (68.57, 72.05)	77.26 (74.81, 79.72)	
Physical activity status			<0.001
<600 MET min/week	15.50 (14.08, 16.92)	23.68 (20.48, 26.87)	
≥600 MET min/week	84.50 (83.08, 85.92)	76.32 (73.13, 79.52)	
Smoke			<0.001
Former	23.53 (22.06, 25.00)	33.73 (31.11, 36.35)	
Never	56.73 (54.53, 58.93)	50.69 (47.36, 54.02)	
Current	19.74 (18.31, 21.17)	15.58 (13.22, 17.95)	
Laboratory tests			
eGFR (mL/min/1.73 m^2^)	98.1 (97.2, 99.0)	72.3 (70.5, 74.0)	<0.001
uACR (mg/g)	7.49 (7.29, 7.69)	191.33 (154.68, 227.99)	<0.001
Fasting triglyceride (mg/dL)	123 (119, 128)	150 (138, 162)	<0.001
Cholesterol (mmol/L)	8.57 (8.54, 8.61)	8.87 (8.79, 8.96)	<0.001
Uric acid (μmol/L)	318 (315, 321)	357 (351, 363)	<0.001
Fasting glucose (mg/dL)	104 (103, 106)	125 (121, 129)	<0.001
Hemoglobin (g/dL)	14.3 (14.2,14.4)	13.9 (13.8, 14.0)	<0.001
Complications			
Hyperuricemia			<0.001
No	84.39 (83.29, 85.49)	65.25 (62.82, 67.68)	
Yes	15.61 (14.51, 16.71)	34.75 (32.32, 37.18)	
Dyslipidemia			<0.001
No	30.18 (28.33, 32.03)	17.48 (15.17, 19.79)	
Yes	69.82 (67.97, 71.67)	82.52 (80.21, 84.83)	
Hypertension			<0.001
No	68.22 (66.42, 70.02)	31.69 (28.51, 34.88)	
Yes	31.78 (29.98, 33.58)	68.31 (65.12, 71.49)	
Diabetes			<0.001
No	89.47 (88.68, 90.26)	63.74 (60.97, 66.50)	
Yes	10.53 (9.74, 11.32)	36.26 (33.50, 39.03)	
Flavonol and subclasses			
Flavonol (mg)	37.72 (36.37, 39.08)	34.78 (31.43, 38.12)	0.096
Isorhamnetin (mg)	1.77 (1.69, 1.85)	1.50 (1.38, 1.61)	<0.001
Kaempferol (mg)	9.62 (9.21, 10.04)	8.31 (7.38, 9.24)	0.011
Myricetin (mg)	3.13 (2.97, 3.29)	3.14 (2.53, 3.75)	0.976
Quercetin (mg)	23.20 (22.37, 24.02)	21.83 (19.96, 23.70)	0.168

Data are expressed as the mean for continuous variables or proportions for categorical variables with adjusted 95% confidence interval; CKD, chronic kidney disease; BMI, body mass index; SBP, systolic blood pressure; DBP, diastolic blood pressure; eGFR, estimated glomerular filtration rate; uACR, urinary albumin-to-creatinine ratio; MET, metabolic equivalent of task.

**Table 2:** Association between dietary flavonol intake and CKD in the US population.

Total flavonols (mg per 48 h)	OR (95%CI)			*p* value for trend
	Model 1	Model 2	Model 3	
Q1 [0, 9.47]	Ref	Ref	Ref	0.190
Q2 (9.47, 18.49]	0.753 (0.599, 0.947)	0.684 (0.541, 0.866)	0.598 (0.349, 1.023)	
Q3 (18.49, 34.66]	0.738 (0.589, 0.923)	0.668 (0.538, 0.831)	0.679 (0.404, 1.142)	
Q4 (34.66, 697.46]	0.622 (0.508, 0.761)	0.554 (0.458, 0.670)	0.628 (0.395, 0.998)	

OR, odds ratio; CI, confidence interval; CKD, chronic kidney disease; Q1, quartile 1; Q2, quartile 2; Q3, quartile 3; Q4, quartile 4.Model 1 did not include any covariate. Model 2 was adjusted for age and sex. Model 3 was adjusted for age, sex, body mass index, ethnicity, smoke status, physical activity status, systolic blood pressure, diastolic blood pressure, energy intake, uric acid, fasting glucose, cholesterol, fasting triglyceride, hyperuricemia, dyslipidemia, hypertension, and diabetes mellitus.

**Table 3:** Association between dietary flavonol intake and decreased eGFR/increased uACR in the US population.

Outcomes	Total flavonol (mg per 48 h)	OR (95%CI)				*p* value for trend
		Q1 [0, 9.47]	Q2 (9.47, 18.49]	Q3 (18.49, 34.66]	Q4 (34.66, 697.46]	
Decreased eGFR		ref	0.479 (0.179, 1.287)	0.372 (0.151, 0.915)	0.665 (0.326, 1.358)	0.703
Elevating uACR		ref	0.589 (0.334, 1.041)	0.747 (0.417, 1.341)	0.520 (0.301, 0.898)	0.060

OR, odds ratio; CI, confidence interval; eGFR, estimated glomerular filtration rate; uACR, urinary albumin-to-creatinine ratio; Q1, quartile 1; Q2, quartile 2; Q3, quartile 3; Q4, quartile 4. Model was adjusted for age, sex, body mass index, ethnicity, smoke status, physical activity status, systolic blood pressure, diastolic blood pressure, energy intake, uric acid, fasting glucose, cholesterol, fasting triglyceride, hyperuricemia, dyslipidemia, hypertension, and diabetes mellitus.

**Table 4:** Examination of the relationship between flavonol intake and CKD in the US population: a stratified analysis by potential modifiers.

Subgroup category	Q1	Q2	Q3	Q4	*p* value for trend	*p* value for interaction
	OR (95%CI)					
Sex						0.413
Female	ref	0.581 (0.349, 0.970)	0.530 (0.307, 0.914)	0.606 (0.360, 1.020)	0.173	
Male	ref	0.736 (0.327, 1.656)	1.000 (0.481, 2.081)	0.780 (0.408, 1.491)	0.688	
Age, years old						0.002
20–59	ref	0.474 (0.207, 1.085)	0.328 (0.150, 0.720)	0.687 (0.366, 1.289)	0.895	
60~	ref	0.685 (0.370, 1.269)	1.105 (0.641, 1.906)	0.630 (0.306, 1.296)	0.345	
Ethnicity						0.377
Black	ref	0.313 (0.097, 1.012)	0.357 (0.133, 0.958)	0.325 (0.115, 0.913)	0.062	
White	ref	0.652 (0.338, 1.258)	0.753 (0.391, 1.451)	0.702 (0.386, 1.274)	0.496	
Mexican	ref	0.402 (0.122, 1.328)	0.320 (0.118, 0.870)	0.134 (0.038, 0.475)	0.003	
Others	ref	1.353 (0.389, 4.712)	1.297 (0.386, 4.356)	1.572 (0.470, 5.253)	0.529	
BMI, kg/m^2^						0.500
≥25	ref	0.683 (0.339, 1.375)	0.696 (0.342, 1.415)	0.662 (0.375, 1.168)	0.251	
<25	ref	0.516 (0.175, 1.522)	0.804 (0.314, 2.058)	0.642 (0.237, 1.735)	0.591	
Smoke status						0.849
Never	ref	0.615 (0.328, 1.154)	0.556 (0.308, 1.005)	0.586 (0.311, 1.105)	0.253	
Former	ref	0.495 (0.197, 1.245)	0.675 (0.254, 1.790)	0.622 (0.286, 1.355)	0.544	
Current	ref	0.888 (0.272, 2.903)	1.384 (0.558, 3.431)	0.713 (0.254, 2.003)	0.540	
Dyslipidemia						0.150
Yes	ref	0.418 (0.220, 0.795)	0.636 (0.336, 1.204)	0.562 (0.337, 0.937)	0.349	
No	ref	2.175 (0.472, 10.033)	0.896 (0.215, 3.741)	1.128 (0.267, 4.772)	0.522	
Hypertension						0.403
Yes	ref	0.472 (0.210, 1.057)	0.456 (0.199, 1.047)	0.516 (0.261, 1.018)	0.211	
No	ref	0.749 (0.378, 1.482)	1.008 (0.447, 2.273)	0.781 (0.358, 1.703)	0.699	
Diabetes mellitus						0.659
Yes	ref	0.475 (0.175, 1.290)	0.426 (0.158, 1.147)	0.582 (0.257, 1.316)	0.513	
No	ref	0.605 (0.318, 1.151)	0.730 (0.429, 1.242)	0.615 (0.366, 1.032)	0.146	
Hyperuricemia						0.872
Yes	ref	0.274 (0.069, 1.084)	0.351 (0.113, 1.086)	0.297 (0.115, 0.771)	0.037	
No	ref	0.734 (0.422, 1.276)	0.839 (0.493, 1.429)	0.767 (0.452, 1.302)	0.577	

OR, odds ratio; CI, confidence interval; BMI, body mass index; Q1, quartile 1; Q2, quartile 2; Q3, quartile 3; Q4, quartile 4.The model was adjusted, if not stratified, for age, sex, body mass index, ethnicity, smoke status, physical activity status, systolic blood pressure, diastolic blood pressure, energy intake, uric acid, fasting glucose, cholesterol, fasting triglyceride, hyperuricemia, dyslipidemia, hypertension, and diabetes mellitus.

**Table 5:** Relationship between the intakes of four flavonol subclasses and chronic kidney disease in US adults.

Flavonol subclasses (mg)	Q1	Q2	Q3	Q4	*p* value for trend
	Adjusted OR (95%CI)				
Isorhamnetin	[0, 0.05]	(0.05, 0.30]	(0.30, 0.80]	(0.80, 75.16]	0.013
	ref	0.860 (0.546,1.354)	0.778 (0.515, 1.177)	0.637 (0.430, 0.943)	
Kaempferol	[0, 0.52]	(0.52, 1.48]	(1.48, 3.82]	(3.82, 152.89]	0.529
	ref	0.840 (0.508, 1.390)	1.030 (0.662, 1.605)	0.817 (0.501, 1.332)	
Myricetin	[0, 0.14]	(0.14, 0.38]	(0.38,1.14]	(1.14,44.75]	0.266
	ref	0.527 (0.300, 0.924)	0.731 (0.408, 1.310)	0.585 (0.315, 1.088)	
Quercetin	[0,3.22]	(3.22, 6.30]	(6.30, 11.38]	(11.38, 202.75]	0.483
	ref	0.632 (0.401, 0.996)	0.798 (0.490, 1.300)	0.736 (0.482, 1.121)	

OR, odds ratio; CI, confidence interval; Q1, quartile 1; Q2, quartile 2; Q3, quartile 3; Q4, quartile 4.All models were adjusted for age, sex, body mass index, ethnicity, smoke status, physical activity status, systolic blood pressure, diastolic blood pressure, energy intake, uric acid, fasting glucose, cholesterol, fasting triglyceride, hyperuricemia, dyslipidemia, hypertension, and diabetes mellitus.

## Results

### Characteristics of the population

Post-weighting, the data in this study represented 213,259,068 American adults aged 20 years and above, with 30,889,883 (14.5%) diagnosed with CKD. As shown in Table 1, there were significant statistical differences between the CKD and non-CKD populations in terms of age, sex, race, blood pressure (both systolic and diastolic), anthropometric measures (height, weight, BMI), lifestyle factors (physical activity status, smoking status, energy intake), renal function markers (eGFR, uACR), lipid profile (fasting triglycerides, total cholesterol), uric acid, fasting glucose, hemoglobin, and prevalence of hyperuricemia, dyslipidemia, hypertension, and diabetes. Additionally, dietary intake of isorhamnetin and kaempferol differed significantly between the groups. Compared to the non-CKD group, the CKD group was older, had a higher proportion of females, higher systolic blood pressure, lower diastolic blood pressure, shorter stature, higher weight, higher BMI, a higher proportion of overweight and obese individuals, a higher proportion of individuals engaging in less than 600 MET min/week of physical activity, lower energy intake, lower eGFR, higher uACR, elevated levels of fasting triglycerides, cholesterol, uric acid and blood glucose, lower hemoglobin, and increased prevalence of hypertension, diabetes, dyslipidemia, and hyperuricemia, along with lower daily intake of isorhamnetin and kaempferol.

### Association between flavonol consumption and CKD

As shown in Table 2, after multivariate adjustment for age, sex, BMI, ethnicity, smoking status, physical activity status, systolic blood pressure, diastolic blood pressure, uric acid, fasting glucose, cholesterol, fasting triglyceride, hyperuricemia, dyslipidemia, hypertension, and diabetes mellitus, the odds ratios (ORs) for CKD risk in the Q2, Q3, and Q4 quartiles of flavonol consumption were 0.598 (95% CI: 0.349, 1.023), 0.679 (95% CI: 0.404, 1.142), and 0.628 (95% CI: 0.395, 0.998), respectively, compared to the Q1 quartile. The *p* value for trend significance was 0.190. As shown in Table 3, after multivariate adjustment, compared to the Q1 quartile, the odds of a decline in eGFR in the Q2, Q3, and Q4 quartiles were 0.479 (95% CI: 0.179, 1.287), 0.372 (95% CI: 0.151, 0.915), and 0.665 (95% CI: 0.326, 1.358), respectively, with a *p* value for trend of 0.703. Furthermore, after multivariate adjustment, compared to quartile Q1, the odds of proteinuria risk in quartiles Q2, Q3, and Q4 were 0.589 (95% CI: 0.334, 1.041), 0.747 (95% CI: 0.417, 1.341), and 0.520 (95% CI: 0.301, 0.898), respectively, with a *p* value for trend significance of 0.060. As shown in Figure 1, RCS analysis revealed that flavonol consumption was non-linearly associated with proteinuria risk and eGFR decline, all with *p* values below 0.05.

### Sensitivity analysis by potential modifiers

The results of our investigation of the relationship between the intake of flavonol from dietary products and the risk of CKD are shown in Table 4. This analysis was divided across different groups, distinguished by factors such as sex, age, racial background, BMI, smoking status, and the presence of conditions like hypertension, diabetes, dyslipidemia, and hyperuricemia. After adjusting for multiple variables, the ORs for CKD incidence in the highest quartile (Q4) compared to the lowest (Q1) were as follows, with 95% confidence intervals (CIs): 0.313 (0.120, 0.815) for Black individuals, 0.338 (0.128, 0.894) for Mexican Americans, 0.562 (0.337,0.937) for dyslipidemia, 0.472 (0.252, 0.885) for hypertension, and 0.297 (0.115,0.771) for those with hyperuricemia. Trend tests for these subgroups mostly showed *p*-values above 0.05, except for Mexican Americans (*p* = 0.005) and those with hyperuricemia (*p* = 0.037). The interaction analysis indicated that for all subgroups, with the exception of the age subgroup, the *p*-values for the interaction tests exceeded 0.05.

### Relationship between intakes of four flavonol subclasses and CKD

**Figure 2:**
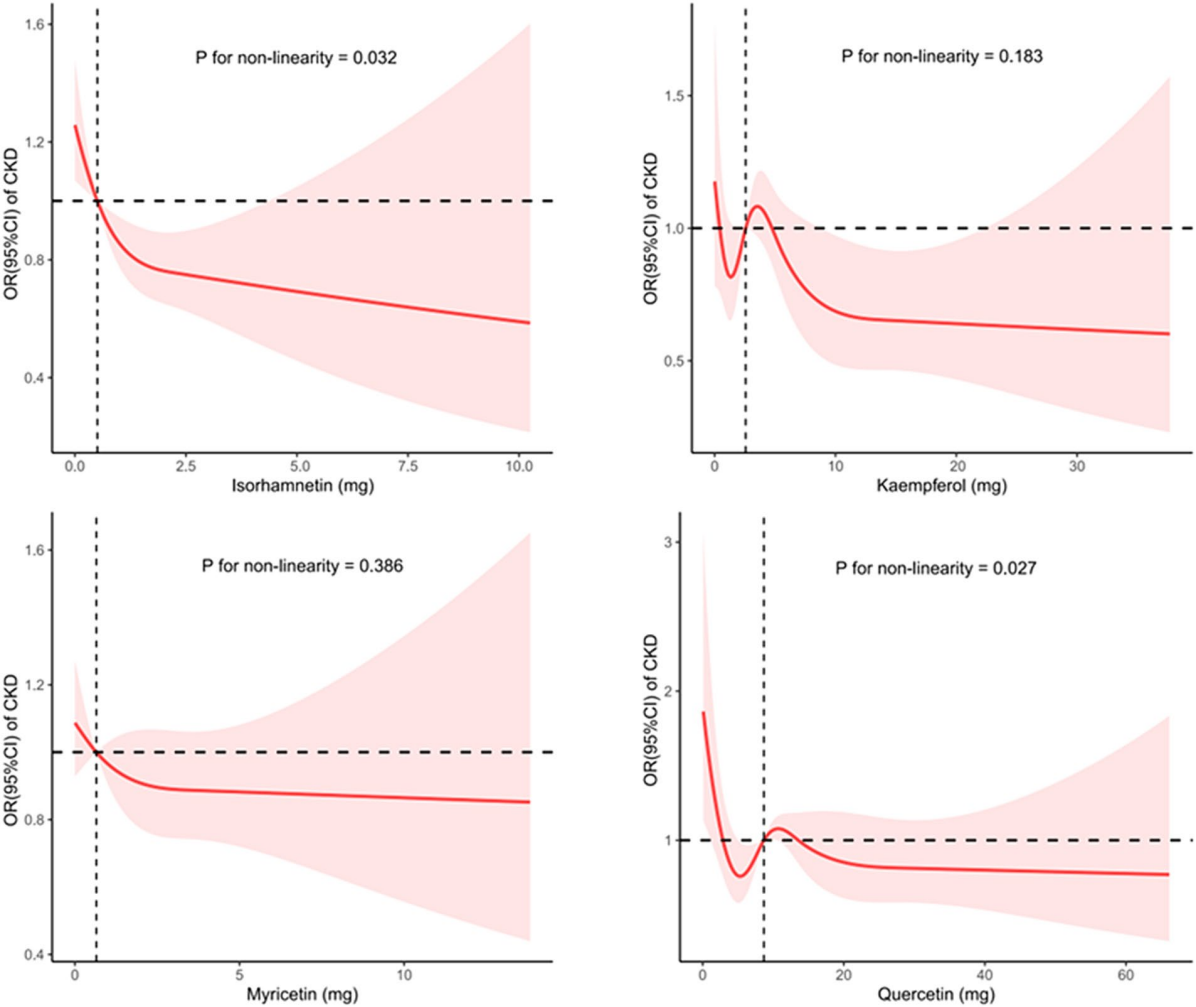
Utilizing restricted cubic splines to analyze the nonlinear trends between the intake of four flavonol subclasses and the risk of CKD.

The dose–response associations of consumption of isorhamnetin, kaempferol, myricetin, and quercetin with CKD are depicted individually in Figure 2, and the association between flavonol intake and CKD in US adults, adjusted for various factors are shown in Table 5. Notably, isorhamnetin intake showed a significant trend, with a reduction in CKD risk (OR: 0.860 [0.546, 1.343] in Q2, OR: 0.778 [0.515, 1.177] in Q3 and 0.637 [0.430, 0.943] in Q4; *p* = 0.013). Kaempferol, myricetin, and querectindid not show significant trends of CKD risk.

## Discussion

Our analysis of the NHANES data from 2007–2010 and 2017–2018 indicates that high consumption of dietary flavonol, especially isorhamnetin, may be associated with a reduced risk of CKD among US adults. This correlation appears to differ among various demographic subgroups and is affected by variables such as age and ethnicity. All models were adjusted for various potential confounding factors, thereby strengthening the reliability of our results.

Existing studies have suggested a potential for the use of flavonols to improve endothelial function, not only in patients with end-stage renal disease and diabetes but also in healthy individuals (11, 25, 26). Considering that endothelial damage plays a role in the development of CKD, it is conceivable that flavonol intake may have protective effects on the kidneys of individuals with CKD (27). This hypothesis is supported by the anti-inflammatory and antioxidant properties of flavonols, which can mitigate the oxidative stress and inflammation associated with CKD progression (19–23). However, a study by Dower et al. did not observe any improvement in endothelial function by quercetin, a type of flavonol (42). On the other hand, a study examining the correlation between diabetic nephropathy and flavonol intake suggested that high flavonoid consumption is linked to a lower incidence of diabetic nephropathy (28). Further analysis revealed that it was flavan-3-ol and flavone, rather than flavonol, which play a critical role in this association. Thus, the potential benefits of flavonol on renal health remain to be determined. In addition, it is also important to acknowledge that the endpoints investigated in the clinical above-mentioned studies differ from those in this study, as they do not pertain to CKD. Our study found a correlation between high flavonol intake and a lower risk of CKD. Noteworthy, although the *p* values for interaction tests were greater than 0.05, variations in CKD incidence relative to flavonol intake were observed across different ethnicities, possibly due to genetic differences, lifestyle variations, and unknown factors.

In the subgroup analysis, we separately examined the associations of quercetin, kaempferol, isorhamnetin, and myricetin consumption with CKD. A previous study showed that quercetin effectively reduces lipopolysaccharide-induced inflammatory responses in human renal tubular epithelial cells (21). Several studies have also shown that quercetin counteracts CKD in mesangial cell models through the regulation of inflammation, oxidative stress, and the transforming growth factor (TGF) related pathway (19, 43). Moreover, it has a significant ability to inhibit asymmetric dimethylarginine-induced apoptosis in glomerular endothelial cells (21). A study by Hsieh et al. revealed that quercetin can reduce DNA damage in the kidneys and restore conditions related to kidney amyloidosis and collagen deposition (44). The studies by Peng and colleagues have shown that, in patients with CKD, quercetin can improve azotemia, reduce hyperuricemia, and relieve the inflammatory response, but it does not reduce serum creatinine levels in mice (45). Additionally, the Fufang Shenhua tablet, a traditional Chinese medicine (TCM) with quercetin as its major component, has been found to improve renal fibrosis (46). *Abelmoschus manihot*, a herbal flowering plant rich in quercetin used in TCM, primarily exerts its therapeutic effect by mitigating inflammatory responses, reducing oxidative stress, and improving renal fibrosis (23). This study failed to establish a clear association between quercetin consumption and the incidence of CKD, potentially as a result of its limited solubility and reduced oral bioavailability (47). Research on the relationship between kaempferol, isorhamnetin, myricetin, and CKD is sparse. Some network-based pharmacological studies suggest that kaempferol and myricetin may have protective effects on renal function, offering new perspectives for the development of new CKD treatment (48, 49). However, this study did not find a correlation between the intake of myricetin or kaempferol and the incidence of CKD, indicating a need for further research to determine the causal relationship between the consumption of kaempferol or myricetin and CKD. Regarding isorhamnetin, some studies found that isorhamnetin can improve TGF-β1-induced glycolysis and renal fibrosis (48, 49). This study found an association between high isorhamnetin intake and a lower incidence of CKD, suggesting that isorhamnetin may benefit CKD patients. Of note, as shown in Figure 2, although the trend test results indicate a statistically significant relationship (*p* value for non-linearity = 0.013), the significance of this correlation disappears after isorhamnetin intake exceeds a certain threshold. This suggests that at higher intake levels, the impact of isorhamnetin on CKD risk is no longer significant. Thus, further basic and clinical research is warranted to confirm this relationship.

This study has several limitations. First, CKD is characterized by impaired kidney function lasting over three months, a long-term condition. In contrast, the evaluation of flavonol intake in this study was based on a 48 h aggregate intake of flavonol, which might not reflect consistent dietary patterns, leading to potential bias in the results. Second, the demographics of the participants in this study were limited to individuals in the US, preventing the generalization of the findings of this study on the effect of flavonol consumption on CKD to other global populations. Third, the lack of renal ultrasound and pathological data might lead to underreporting of CKD cases, thus introducing a possible bias. Fourth, the study did not account for the possibility that high daily intake of quercetin could interfere with the effects of other flavonol compounds.

## Conclusion

In our analysis of the data from the NHANES periods 2007–2008, 2009–2010, and 2017–2018, we found a possible negative association between increased consumption of total flavonol, especially isorhamnetin, and the risk of CKD. This finding could pave the way for the development of innovative approaches for the treatment of CKD. However, the establishment of this association between flavonol consumption and CKD incidence requires additional verification using a more comprehensive cohort and randomized controlled trials.

## Data availability statement

Publicly available datasets were analyzed in this study. This data can be found here: https://www.cdc.gov/nchs/nhanes.

## Ethics statement

The studies involving humans were approved by National Center for Health Statistics Ethics Review Board. The studies were conducted in accordance with the local legislation and institutional requirements. The participants provided their written informed consent to participate in this study.

## Author contributions

PL: Conceptualization, Data curation, Formal analysis, Investigation, Software, Writing – original draft. LT: Conceptualization, Writing – original draft. GL: Formal analysis, Funding acquisition, Writing – original draft. XW: Conceptualization, Writing – original draft. FH: Writing – review & editing. WP: Writing – review & editing.

## References

[1] GBD Chronic Kidney Disease Collaboration. Global, regional, and national burden of chronic kidney disease, 1990–2017: a systematic analysis for the global burden of disease study 2017. Lancet. (2020) 395:709–33. doi: 10.1016/S0140-6736(20)30045-3 32061315 PMC7049905

[2] Centers for Disease Control and Prevention. Chronic kidney disease in the United States, 2023. Atlanta, GA: US Department of Health and Human Services, Centers for Disease Control and Prevention (2023).

[3] Chen TK Knicely DH Grams ME. Chronic kidney disease diagnosis and management: a review. JAMA. (2019) 322:1294–304. doi: 10.1001/jama.2019.14745, PMID: 31573641 PMC7015670

[4] Inker LA Astor BC Fox CH Isakova T Lash JP Peralta CA. KDOQI US commentary on the 2012 KDIGO clinical practice guideline for the evaluation and management of CKD. Am J Kidney Dis. (2014) 63:713–35. doi: 10.1053/j.ajkd.2014.01.416, PMID: 24647050

[5] KDIGO. Clinical practice guideline for the Management of Glomerular Diseases. Kidney Int. (2021) 100:S1–s276. doi: 10.1016/j.kint.2021.05.021 34556256

[6] Bangar SP Chaudhary V Sharma N Bansal V Ozogul F Lorenzo JM. Kaempferol: a flavonoid with wider biological activities and its applications. Crit Rev Food Sci Nutr. (2023) 63:9580–604. doi: 10.1080/10408398.2022.2067121, PMID: 35468008

[7] Jucá MM Cysne Filho FMS de Almeida JC Mesquita DDS Barriga JRM Dias KCF. Flavonoids: biological activities and therapeutic potential. Nat Prod Res. (2020) 34:692–705. doi: 10.1080/14786419.2018.1493588 30445839

[8] Shen N Wang T Gan Q Liu S Wang L Jin B. Plant flavonoids: classification, distribution, biosynthesis, and antioxidant activity. Food Chem. (2022) 383:132531. doi: 10.1016/j.foodchem.2022.132531, PMID: 35413752

[9] Wen L Jiang Y Yang J Zhao Y Tian M Yang B. Structure, bioactivity, and synthesis of methylated flavonoids. Ann N Y Acad Sci. (2017) 1398:120–9. doi: 10.1111/nyas.13350 28436044

[10] Ottaviani JI Heiss C Spencer JPE Kelm M Schroeter H. Recommending flavanols and procyanidins for cardiovascular health: revisited. Mol Asp Med. (2018) 61:63–75. doi: 10.1016/j.mam.2018.02.001, PMID: 29427606

[11] Balzer J Rassaf T Heiss C Kleinbongard P Lauer T Merx M. Sustained benefits in vascular function through flavanol-containing cocoa in medicated diabetic patients a double-masked, randomized, controlled trial. J Am Coll Cardiol. (2008) 51:2141–9. doi: 10.1016/j.jacc.2008.01.059, PMID: 18510961

[12] Holland TM Agarwal P Wang Y Dhana K Leurgans SE Shea K. Association of Dietary Intake of Flavonols with changes in global cognition and several cognitive abilities. Neurology. (2023) 100:e694–702. doi: 10.1212/WNL.0000000000201541, PMID: 36414424 PMC9969915

[13] Dong X Zhou S Nao J. Kaempferol as a therapeutic agent in Alzheimer’s disease: evidence from preclinical studies. Ageing Res Rev. (2023) 87:101910. doi: 10.1016/j.arr.2023.101910, PMID: 36924572

[14] Guo XF Ruan Y Li ZH Li D. Flavonoid subclasses and type 2 diabetes mellitus risk: a meta-analysis of prospective cohort studies. Crit Rev Food Sci Nutr. (2019) 59:2850–62. doi: 10.1080/10408398.2018.1476964, PMID: 29768032

[15] Sahebkar A. Effects of quercetin supplementation on lipid profile: a systematic review and meta-analysis of randomized controlled trials. Crit Rev Food Sci Nutr. (2017) 57:666–76. doi: 10.1080/10408398.2014.948609 25897620

[16] Zhao Z Gao W Ding X Xu X Xiao C Mao G. The association between dietary intake of flavonoids and its subclasses and the risk of metabolic syndrome. Front Nutr. (2023) 10:1195107. doi: 10.3389/fnut.2023.1195107, PMID: 37476404 PMC10354435

[17] Tong J Zeng Y Xie J Xiao K Li M Cong L. Association between flavonoid and subclasses intake and metabolic associated fatty liver disease in U.S. adults: Results from National Health and Nutrition Examination Survey 2017–2018. Front Nutr. (2022) 9:1074494. doi: 10.3389/fnut.2022.1074494, PMID: 36532560 PMC9751205

[18] Oei S Millar CL Nguyen Lily TN Mukamal KJ Kiel DP Lipsitz LA. Higher intake of dietary flavonols, specifically dietary quercetin, is associated with lower odds of frailty onset over 12 years of follow-up among adults in the Framingham heart study. Am J Clin Nutr. (2023) 118:27–33. doi: 10.1016/j.ajcnut.2023.04.013 37061164 PMC10447475

[19] Widowati W Prahastuti S Tjokropranoto R Onggowidjaja P Kusuma HSW Afifah E. Quercetin prevents chronic kidney disease on mesangial cells model by regulating inflammation, oxidative stress, and TGF-β1/SMADs pathway. PeerJ. (2022) 10:e13257. doi: 10.7717/peerj.13257 35673387 PMC9167587

[20] Ren Q Tao S Guo F Wang B Yang L Ma L. Natural flavonol fisetin attenuated hyperuricemic nephropathy via inhibiting IL-6/JAK2/STAT3 and TGF-β/SMAD3 signaling. Phytomedicine. (2021) 87:153552. doi: 10.1016/j.phymed.2021.153552, PMID: 33994251

[21] Guo S Sun J Zhuang Y. Quercetin alleviates lipopolysaccharide-induced inflammatory responses by up-regulation miR-124 in human renal tubular epithelial cell line HK-2. Biofactors. (2020) 46:402–10. doi: 10.1002/biof.1596, PMID: 31804760

[22] Wang F Zhao X Su X Song D Zou F Fang L. Isorhamnetin, the xanthine oxidase inhibitor from Sophora japonica, ameliorates uric acid levels and renal function in hyperuricemic mice. Food Funct. (2021) 12:12503–12. doi: 10.1039/D1FO02719K, PMID: 34806108

[23] Wei C Wang C Li R Bai Y Wang X Fang Q. The pharmacological mechanism of Abelmoschus manihot in the treatment of chronic kidney disease. Heliyon. (2023) 9:e22017. doi: 10.1016/j.heliyon.2023.e22017, PMID: 38058638 PMC10695975

[24] Rassaf T Rammos C Hendgen-Cotta UB Heiss C Kleophas W Dellanna F. Vasculoprotective effects of dietary cocoa Flavanols in patients on hemodialysis: a double-blind, randomized, placebo-controlled trial. Clin J Am Soc Nephrol. (2016) 11:108–18. doi: 10.2215/CJN.05560515, PMID: 26681132 PMC4702234

[25] Sansone R Rodriguez-Mateos A Heuel J Falk D Schuler D Wagstaff R. Cocoa flavanol intake improves endothelial function and Framingham risk score in healthy men and women: a randomised, controlled, double-masked trial: the Flaviola health study. Br J Nutr. (2015) 114:1246–55. doi: 10.1017/S0007114515002822, PMID: 26348767 PMC4594054

[26] Rodriguez-Mateos A Hezel M Aydin H Kelm M Lundberg JO Weitzberg E. Interactions between cocoa flavanols and inorganic nitrate: additive effects on endothelial function at achievable dietary amounts. Free Radic Biol Med. (2015) 80:121–8. doi: 10.1016/j.freeradbiomed.2014.12.009, PMID: 25530151

[27] Vargas F Romecín P García-Guillén AI Wangesteen R Vargas-Tendero P Paredes MD. Flavonoids in kidney health and disease. Front Physiol. (2018) 9:394. doi: 10.3389/fphys.2018.00394, PMID: 29740333 PMC5928447

[28] Liu F Nie J Deng MG Yang H Feng Q Yang Y. Dietary flavonoid intake is associated with a lower risk of diabetic nephropathy in US adults: data from NHANES 2007–2008, 2009–2010, and 2017–2018. Food Funct. (2023) 14:4183–90. doi: 10.1039/D3FO00242J, PMID: 37066968

[29] Zipf G Chiappa M Porter KS Ostchega Y Lewis BG Dostal J. National Health and Nutrition Examination Survey: plan and operations, 1999–2010. Vital Health Stat. (2013) 1:1–37. 25078429

[30] Sebastian RS Wilkinson Enns C Goldman JD Martin CL Steinfeldt LC Murayi T. A new database facilitates characterization of flavonoid intake, sources, and positive associations with diet quality among US adults. J Nutr. (2015) 145:1239–48. doi: 10.3945/jn.115.213025, PMID: 25948787 PMC4442120

[31] Rhodes DG Murayi T Clemens JC Baer DJ Sebastian RS Moshfegh AJ. The USDA automated multiple-pass method accurately assesses population sodium intakes. Am J Clin Nutr. (2013) 97:958–64. doi: 10.3945/ajcn.112.044982, PMID: 23553153

[32] Moshfegh AJ Rhodes DG Baer DJ Murayi T Clemens JC Rumpler WV. The US Department of Agriculture Automated Multiple-Pass Method reduces bias in the collection of energy intakes. Am J Clin Nutr. (2008) 88:324–32. doi: 10.1093/ajcn/88.2.324, PMID: 18689367

[33] Chavers BM Simonson J Michael AF. A solid phase fluorescent immunoassay for the measurement of human urinary albumin. Kidney Int. (1984) 25:576–8. doi: 10.1038/ki.1984.57, PMID: 6737844

[34] Masson P Ohlsson P Björkhem I. Combined enzymic-Jaffé method for determination of creatinine in serum. Clin Chem. (1981) 27:18–21. doi: 10.1093/clinchem/27.1.18, PMID: 7449105

[35] Inker LA Schmid CH Tighiouart H Eckfeldt JH Feldman HI Greene T. Estimating glomerular filtration rate from serum creatinine and cystatin C. N Engl J Med. (2012) 367:20–9. doi: 10.1056/NEJMoa1114248 22762315 PMC4398023

[36] Ali MK Bullard KM Beckles GL Stevens MR Barker L Narayan KM. Household income and cardiovascular disease risks in U.S. children and young adults: analyses from NHANES 1999–2008. Diabetes Care. (2011) 34:1998–2004. doi: 10.2337/dc11-0792 21868776 PMC3161277

[37] Vilar-Gomez E Nephew LD Vuppalanchi R Gawrieh S Mladenovic A Pike F. High-quality diet, physical activity, and college education are associated with low risk of NAFLD among the US population. Hepatology. (2022) 75:1491–506. doi: 10.1002/hep.32207, PMID: 34668597

[38] Valsaraj R Singh AK Gangopadhyay KK Ghoshdastidar B Goyal G Batin M. Management of asymptomatic hyperuricemia: Integrated Diabetes & Endocrine Academy (IDEA) consensus statement. Diabetes Metabolic Syndrome. (2020) 14:93–100. doi: 10.1016/j.dsx.2020.01.007, PMID: 31991299

[39] Arvanitis M Lowenstein CJ. Dyslipidemia. Ann Intern Med. (2023) 176:Itc81. doi: 10.7326/AITC202306200 37307585

[40] Classification and Diagnosis of Diabetes. Standards of medical Care in Diabetes-2021. Diabetes Care. (2021) 44:S15–s33. doi: 10.2337/dc21-S002 33298413

[41] Messerli FH Williams B Ritz E. Essential hypertension. Lancet. (2007) 370:591–603. doi: 10.1016/S0140-6736(07)61299-9 17707755

[42] Dower JI Geleijnse JM Gijsbers L Zock PL Kromhout D Hollman PC. Effects of the pure flavonoids epicatechin and quercetin on vascular function and cardiometabolic health: a randomized, double-blind, placebo-controlled, crossover trial. Am J Clin Nutr. (2015) 101:914–21. doi: 10.3945/ajcn.114.098590, PMID: 25934864

[43] Cao Y Hu J Sui J Jiang L Cong Y Ren G. Quercetin is able to alleviate TGF-β-induced fibrosis in renal tubular epithelial cells by suppressing miR-21. Exp Ther Med. (2018) 16:2442–8. doi: 10.3892/etm.2018.6489, PMID: 30210596 PMC6122524

[44] Hsieh CL Peng CC Chen KC Peng RY. Rutin (quercetin rutinoside) induced protein-energy malnutrition in chronic kidney disease, but quercetin acted beneficially. J Agric Food Chem. (2013) 61:7258–67. doi: 10.1021/jf304595p, PMID: 23876017

[45] Peng CC Hsieh CL Ker YB Wang HY Chen KC Peng RY. Selected nutraceutic screening by therapeutic effects on doxorubicin-induced chronic kidney disease. Mol Nutr Food Res. (2012) 56:1541–58. doi: 10.1002/mnfr.201200178 22945467

[46] Li R Shi C Wei C Wang C Du H Liu R. Fufang Shenhua tablet inhibits renal fibrosis by inhibiting PI3K/AKT. Phytomedicine. (2023) 116:154873. doi: 10.1016/j.phymed.2023.154873, PMID: 37257328

[47] Chen YQ Chen HY Tang QQ Li YF Liu XS Lu FH. Protective effect of quercetin on kidney diseases: from chemistry to herbal medicines. Front Pharmacol. (2022) 13:968226. doi: 10.3389/fphar.2022.968226, PMID: 36120321 PMC9478191

[48] Xie X Lou H Shi Y Gan G Deng H Ma X. A network pharmacological-based study of the mechanism of Liuwei Dihuang pill in the treatment of chronic kidney disease. Medicine. (2023) 102:e33727. doi: 10.1097/MD.0000000000033727, PMID: 37171332 PMC10174353

[49] Sun X Huang Y Zhu S Yan J Gan K Xu Z. Yishen Qingli Heluo granule in the treatment of chronic kidney disease: network pharmacology analysis and experimental validation. Drug Des Devel Ther. (2022) 16:769–87. doi: 10.2147/DDDT.S348335, PMID: 35355655 PMC8959874

